# High patient satisfaction and increased physical activity following a remote multidisciplinary team multiple myeloma clinic

**DOI:** 10.1007/s00520-023-07587-9

**Published:** 2023-01-21

**Authors:** Catherine S. Y. Lecat, Abigail Fisher, Maria Atta, Marquita Camilleri, Orla McCourt, Joanne Land, Sarah Worthington, Alyse Hart, Angela Daniel, Inayah Uddin, Charlotte Roche, Holger W. Auner, Kwee Yong

**Affiliations:** 1 University College London Cancer Institute, 72 Huntley Street, London, WC1E 6DD UK; 2 University College London Institute of Epidemiology & Health Care, 1-19 Torrington Place, London, WC1E 7HB UK; 3 Imperial College Healthcare NHS Trust, Hammersmith Hospital, 72 Du Cane Road, London, W12 0HS UK; 4 University College London Hospitals NHS Foundation Trust, 235 Euston Road, London, NW1 2BU UK; 5 The Hugh and Josseline Langmuir Centre for Myeloma Research, Imperial College London, Du Cane Road, London, W12 0NN UK

**Keywords:** Multiple myeloma, Multidisciplinary team, PrISMS clinic

## Abstract

**Purpose:**

Patients with multiple myeloma suffer from disease-related complications such as bone destruction, toxicities from repeated therapies and age-related co-morbidities. With improved treatment options, patients are living longer and have specific survivorship needs such as low exercise levels that need to be addressed. In this study, we designed, implemented and evaluated a multidisciplinary team (MDT) myeloma clinic that provided participants with tailored exercise and lifestyle advice.

**Methods:**

The Promoting Individualised Self-Management and Survivorship (PrISMS) clinic was set up in two UK myeloma centres. This remote MDT clinic comprised of a doctor, a nurse specialist and a physiotherapist. Patients were required to complete blood tests and a questionnaire about their symptoms and concerns before each consultation. Patient-reported outcome measures were captured using validated questionnaires. Patient feedback was collected using a specially designed survey and structured telephone interviews.

**Results:**

Sixty-one patients were enrolled in the pilot clinic with 210 consultations held during the study period. Nine patients had disease progression and were referred safely back to face-to-face clinics. There was a significant improvement in patients’ exercise score (*p* = 0.02) after PrISMS clinic. Patient satisfaction was high, with 83% feeling more confident in self-managing myeloma after PrISMS clinic.

**Conclusion:**

PrISMS clinic is safe and feasible, with high patient compliant and acceptability. It empowers patients to self-manage their condition and encourages physical activity, which is associated with improved quality of life and fatigue level. Future randomised controlled trials will help to confirm its benefits on patient clinical outcomes and cost-effectiveness.

**Supplementary Information:**

The online version contains supplementary material available at 10.1007/s00520-023-07587-9.

## Introduction

Multiple myeloma (MM) is an incurable bone marrow malignancy characterised by a specific pattern of end-organ damage, including bone destruction, anaemia, hypercalcaemia and renal impairment. It is more common in older adults, and patients usually go through periods of remission and relapse, followed by eventual treatment-resistant disease [1]. Even during disease remission, patients often continue to struggle with myeloma-related long-term complications such as bone destruction resulting in spinal deformity and reduced mobility, as well as treatment toxicities such as neuropathy and age-related co-morbidities [2]. All of these factors contribute to slow recovery of fitness and impede return to work and normal social activities [3], which inevitably impact on quality of life [2, 4, 5]. Patient survival has improved significantly with therapeutic advances, but this has also resulted in an increasing number of patients in outpatient haematology clinics with specific survivorship needs. Our group previously conducted a survey involving 78 patients and identified that patients would like lifestyle support and advice in order to return to their pre-morbid social, psychological and economic functionality.

Physical activity (PA) has been shown to associate with lower fatigue, improved health-related quality of life (HRQOL) and physical functioning in cancer patients, with some evidence in MM [6, 7]. With a high proportion of MM patients suffering from bone destruction [8], some may be fearful to exercise despite its benefits and require professional supervision to promote this safely [9, 10]. Our previous work revealed that most myeloma patients had low self-reported PA levels and high fatigue levels [11]. We demonstrated an association between higher exercise levels and better HRQOL, as well as lower fatigue levels. Majority of participants would like to improve their level of PA and to receive PA advice from a healthcare professional. This suggests an awareness of the possible benefits of exercise and an unmet survivorship need [11]. Furthermore, our group demonstrated that exercise programmes were safe for myeloma survivors, with objective health benefits including improved muscle strength, cardiovascular fitness and fatigue [9, 10].

Although patients value and benefit from PA advice, centralisation of transplant and clinical trial services means that many patients travel long distances to maintain contact with their myeloma specialist centres. Furthermore, the COVID-19 pandemic has resulted in drastic changes in the structure of outpatient services in order to reduce infection risk [12]. There has been a shift towards remote consultations where possible, particularly with vulnerable patient groups including those with cancer. Therefore, there is a need to develop a more holistic outpatient care model that is patient-centred with personalised lifestyle and exercise advice given to MM patients and is delivered in a way that is acceptable and geographically convenient for them whilst maintaining contact with specialist centres. The aims of this study were to develop and test such a model and to evaluate its safety and feasibility as primary outcomes, with patient acceptability and its effects on patient-reported outcome measures (PROMs) as secondary outcomes.

## Methods

### The Promoting Individualised Self-Management and Survivorship myeloma clinic

**Fig. 1:**
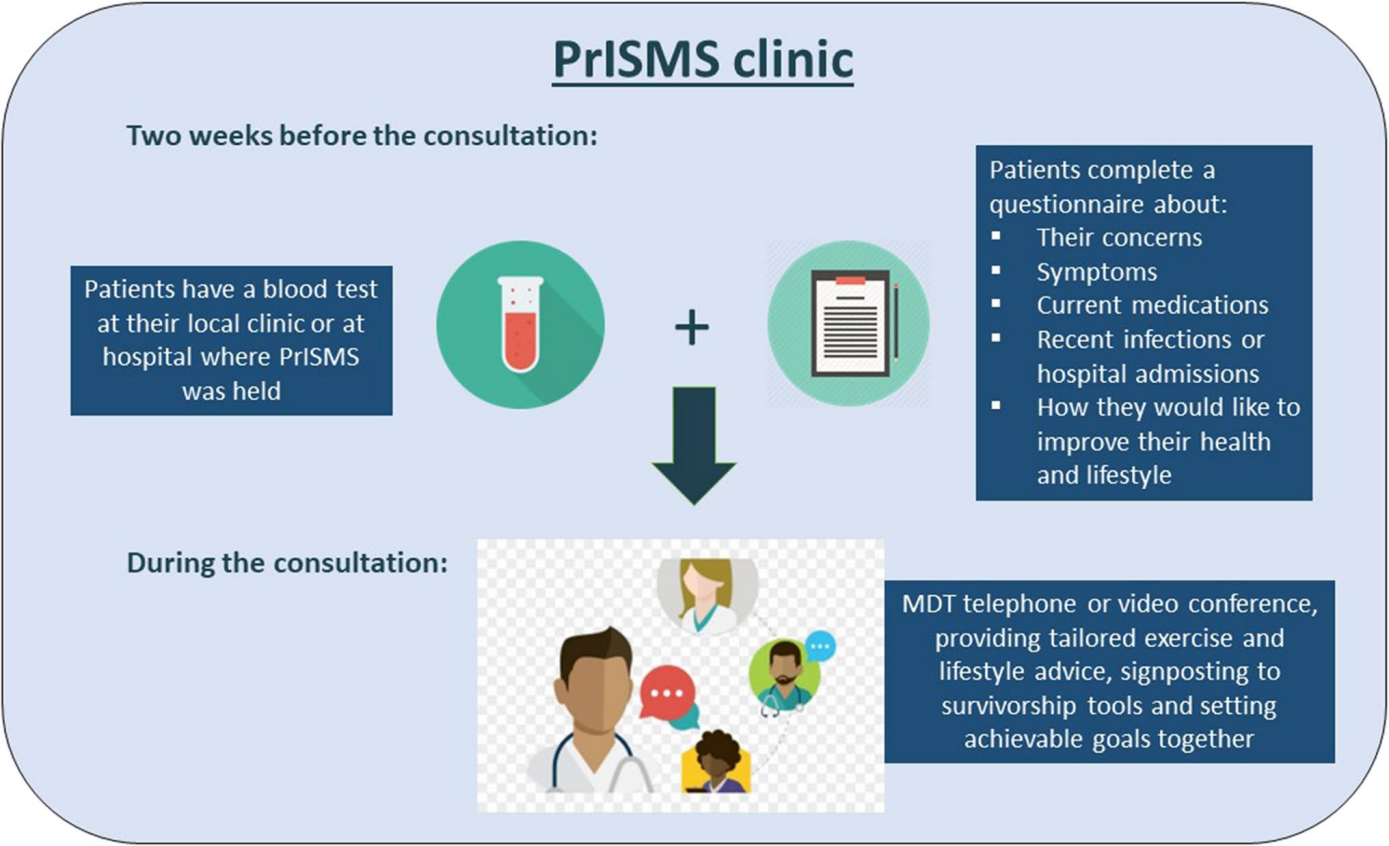
The PrISMS clinic design

The Promoting Individualised Self-Management and Survivorship (PrISMS) clinic was designed for this study. This remote clinic was staffed by a doctor, a cancer nurse specialist (CNS) and a physiotherapist (PT), with wider multidisciplinary team (MDT) support (e.g. dieticians, psychologists, social welfare workers) if needed. It aimed to provide holistic management centred on patient needs through giving individualised PA and lifestyle advice (Fig. 1). Two weeks before the consultation, patients were invited via email to complete a pre-clinic questionnaire called ‘My Myeloma Review’ about their concerns, symptoms and ways in which they would like to improve their health and lifestyle (see Supplementary information). Returned questionnaires allowed the MDT to prepare appropriate advice for each patient, ensuring efficient use of consultations. They were also required to have a blood test (see details in Supplementary information) 2 weeks before and could choose to do this at their local hospital or general practice (GP) surgery, or at the hospital where the PrISMS clinic was held. Results of blood tests were essential for monitoring patients’ health and disease activity. If CNS or PT was unable to attend the clinic, patients were offered to be contacted by them at a later time point. Those with complex physiotherapy needs were offered additional remote or F2F sessions with the physiotherapist. To participate in this study, patients had to be ≥ 18 years, understood and spoke fluent English, in remission or plateau phase and not on any active myeloma treatment, did not have any features that were deemed unsuitable for remote monitoring (e.g. previous rapidly progressing disease, high-risk cytogenetics requiring close monitoring) and were able to give informed consent. This study was approved by the Research Ethics Committee of the NHS Health Research Authority (Reference: 19/NS/0105).

### Data collection from pilot PrISMS clinics

Logistical data such as call duration, patient and MDT attendance were recorded. To collect PROMs, participants were asked to complete validated questionnaires before their first and after their last PrISMS appointment during the research period: the Godin Leisure-Time Exercise Questionnaire (GLTEQ) [13, 14] to measure PA level, the European Organisation for Research and Treatment of Cancer (EORTC) QLQ-C30 questionnaire to assess HRQOL [15] and its myeloma module MY20 to determine disease-specific symptoms and concerns [16]. The Myeloma Patient Outcome Scale (MyPOS) was used to assess palliative care concerns, information needs and healthcare support [17]. Missing responses on questionnaires were handled according to EORTC scoring manuals, and for MyPOS, relevant domains were excluded from analysis if there were > 2 missing responses in that domain. These questionnaires were screened for clinically important issues, which would be fed back to the clinical team.

Patient feedback was collected through a written questionnaire that was specially designed to understand their views on clinic design and their experience. Semi-structured telephone interviews were also conducted by researchers with a background in Health Psychology, who were not involved in participants’ myeloma care. Interviews were conducted by telephone. Recorded audio files were stored securely without patient-identifiable data and were transcribed for analysis. Feedback questionnaire and interview topic guide are available in the Supplementary information.

### Data analyses

Quantitative data were presented as percentages, mean, median and quartiles. Non-parametric Wilcoxon test was used to compare paired results. A *p*-value of < 0.05 was considered statistically significant. SPSS was used to perform statistical analyses. Qualitative data from patient interviews were analysed using the Framework Method [18].

## Results

### PrISMS patients and their consultations

Between March 2019 and October 2020, two PrISMS clinics were set up at University College London Hospital (UCLH) and Imperial College Healthcare NHS Trust (ICHNT), which were both myeloma tertiary centres; 61 patients were enrolled (54 at UCLH and 7 at ICHNT). Patient characteristics are shown in Table 1; 89% had a previous autologous stem cell transplant (ASCT). Of the 61 patients, 12 (20%) were referred back to face-to-face (F2F) clinic (3 due to patient’s preference, 9 due to disease progression), 7 (11%) were discharged back to their local hospital in accordance with their preference and 1 died of non-myeloma cause; 41 remained in PrISMS clinic at the end of the research period. All 9 patients who had disease progression were referred safely and promptly back to the F2F clinic: 2 had a rapid biochemical relapse on blood tests and 7 had evidence of disease progression on scans that were requested due to new symptoms.

**Table 1:** Patient characteristics

Patient characteristics (*n* = 61)		Frequency (percentage %)
Gender	Male	40 (66)
	Female	21 (34)
Age	< 45	4 (7)
	45–55	10 (16)
	56–65	21 (34)
	66–75	26 (43)
Immunoglobulin isotype	IgG	33 (54)
	IgA	7 (11)
	Light chain MM	15 (25)
	Plasmacytoma only	6 (10)
Light chain isotype	Kappa	38 (62)
	Lambda	18 (30)
	Not applicable	4 (7)
	Missing data	1 (2)
International Myeloma working group cytogenetic risk^a^	Standard risk	24 (39)
	High risk	11 (18)
	Not known	26 (43)
Prior lines of myeloma therapy	1	45 (74)
	2	12 (20)
	3	3 (5)
	4	1 (2)
Prior autologous stem cell transplant	Yes	54 (89)
	No	7 (11)
Number of PrISMS consultations attended per patient	1	12 (20)
	2	14 (23)
	3	3 (5)
	4	11 (18)
	5	14 (23)
	6	6 (10)
	8	1 (2)

^a^International Myeloma Working Group (IMWG) Molecular Classification of Multiple Myeloma. Available at https://www.myeloma.org/resource-library/international-myeloma-working-group-imwg-molecular-classification-multiple-myeloma

210 telephone or video consultations were held (197 at UCLH, 13 at ICHNT), with a median call duration of 13 min (range 2–35). There was one missed consultation (0.5%), and 6 were postponed (2.4%; 1 administrative error, 1 patient abroad, 1 concurrent illness, 3 delays in organising blood test locally). Patient compliance was high, with pre-clinic blood tests and ‘My Myeloma Review’ questionnaires completed in 89% (187/210) and 85% (178/210) of the consultations, respectively.

### Patient feedback

#### Patient feedback on pre-clinic blood test and ‘My Myeloma Review’ questionnaire

Forty-two patients (69% of 61 enrolled) completed the feedback questionnaire. Regarding pre-clinic blood test, all patients agreed (11/42, 26%) or strongly agreed (31/42, 74%) that it was useful having blood results available at the time of PrISMS consultation (Supplementary Fig. 1). In general, there was no issue in arranging blood tests at UCLH and ICHNT, though the accessibility of blood tests locally outside of these centres varied depending on where patients lived. Several commented on the difficulty in organising local blood tests due to increased demand or restrictions as a result of COVID-19.

The majority of patients agreed (23/42, 55%) or strongly agreed (10/42, 24%) that the pre-clinic ‘My Myeloma Review’ questionnaire was useful in monitoring their own health. The remaining patients (9/42, 21%) neither agreed nor disagreed; 91% (38/42) agreed or strongly agreed that it was easy to complete, with 9% (4/42) neither agreed nor disagreed (Supplementary Fig. 1); 81% (34/42) felt that the questionnaire improved the quality of their clinic appointment. Patients also commented on how it guided them through what information was expected from them during the clinic, and that it allowed clinicians to be well briefed before the consultation so that they could get to the major points quickly. One patient preferred the questionnaire to be sent by post instead, and another would like it to be available via a web link for ease of completion.

#### Patient feedback on PT and CNS input

PT attended 64% (135/210) of the consultations and were actively involved in 53% of them (112/210); 90% (55/61) of the patients received PT input at any point in time. Examples of PT input include providing individually tailored exercise programmes, signposting or referring patients to local exercise schemes, and sending exercise leaflets or weblinks to promote independent exercise; 11 patients (18%) were referred to a local exercise scheme. Supplementary Table 1 shows examples of PA and other survivorship tools that patients were signposted to. Results from the feedback questionnaire showed that 93% (39/42) of patients recalled discussing PA during their consultations; 57% (24/42) agreed or strongly agreed that having a PT in the clinic was helpful; 29% (12/42) neither agreed nor disagreed, 12% (5/42) did not specify, and one patient strongly disagreed with this (Supplementary Fig. 2). Four patients mentioned in the questionnaire that the physiotherapy advice that they received had helped to relieve their existing pain and discomfort.

CNS attended 76% (160/210) of the consultations and were actively involved in 43% of them (91/210); 80% (49/61) of participants received CNS input at any point in time. Examples of CNS input include giving information on MM, dietary advice, psychological support, financial and welfare advice, discussing re-vaccination after ASCT, smoking cessation and alcohol consumption (Supplementary Table 1); 3 patients (5%) were referred to psychological counselling service; 71% (30/42) agreed or strongly agreed in the feedback questionnaire that having a CNS in the consultation was helpful; 26% (11/42) neither agreed nor disagreed, and one patient did not find it helpful (Supplementary Fig. 2). One patient mentioned that it was good to have the opportunity to discuss work-related issues in this MDT clinic.

#### Patient feedback on PrISMS clinic experience

**Fig. 2:**
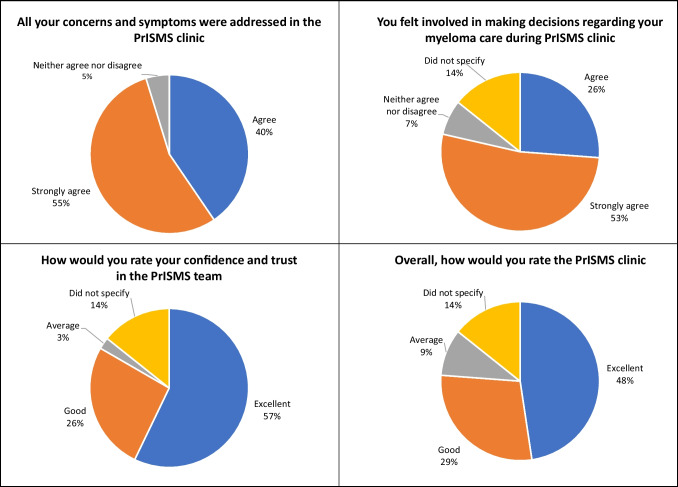
Feedback from patient surveys regarding their experience in the PrISMS clinic

When asked in the feedback questionnaire about whether their concerns and symptoms were addressed in the PrISMS clinic, 40% (17/42) agreed, 55% (23/42) strongly agreed and two patients neither agreed nor disagreed. Most patients felt that they were involved in making decisions in their myeloma care (78% agreed or strongly agreed) and felt more confident in self-managing their myeloma after the consultation (83% agreed or strongly agreed). When asked to rate their confidence and trust in the PrISMS team via remote consultations, 57% (24/42) rated excellent, 26% (11/42) rated good, with one patient (2%) rated average. Overall ratings for PrISMS compared to the F2F clinic were also positive, with 47% (20/42) rated excellent, 29% (12/42) rated good and 10% (4/42) rated average (Fig. 2).

Additional comments from the feedback questionnaires include the benefits of saving time, energy and cost by not having to travel to the hospital with this remote clinic. It also reduced anxiety that could occur when waiting physically in the hospital to see a doctor. Some expressed that they would still prefer to be reviewed occasionally in a F2F clinic, where they could be examined and assessed properly. One patient mentioned that PrISMS clinic worked well because he had met the team physically before. In addition, several patients felt that remote monitoring was appropriate when they had no clinical issues, but this might change if they had felt unwell or had relapsed disease.

#### Semi-structured telephone interviews

Twenty-two patients were interviewed, and themes and quotes are shown in Table 2. In addition to the comments in the feedback questionnaires, some patients found the pre-clinic ‘My Myeloma Review’ questionnaire helpful as it covered both mental and physical well-being topics. Others felt that it may not be suited for asymptomatic patients with few concerns, but nonetheless a useful tool to prompt more holistic discussion with clinicians. It was noted by two patients that the questionnaire did not address sexual activity.

**Table 2:** Examples of patient quotes from telephone interviews

Examples of patient quotes from telephone interviews	
Pre-clinic blood test and questionnaire	*“With the PrISMS process I get it (blood result) there and then and that's actually really quite a big weight off my mind… So, in that respect it's been super-helpful really, a big improvement.”* *“For my wife and I to come to London, we have to lay out £50. It is so much nicer to have blood tests done locally and an hour later I could be having a normal day.”* *“The crib (questionnaire) is a nice idea because it is a reminder. It makes you review what you wouldn’t otherwise do. And I don’t think I would have done if I had a face-to-face consultation.”* *“(The questionnaire) allows me to direct the doctors to talk about the things that I want to talk about. Sometimes when you have a phone interview, you don’t get to say the things you want to say, it gives the chance to set the agenda for the discussion.”*
Waiting and travel time	*“It saves me, what, the best part of two hours travelling, there and back. But also because, the feeling that I got was the (PrISMS) consultations are much more likely to happen roughly at the right time, compared with face-to-face consultations, which could be an hour and a half later.”* *“People would sit around waiting, waiting for ages in hospital, for their clinic appointment, and it’s always running late. I’ve seen people in tears, because it is a very stressful thing.”*
Remote clinic consultation	*“I think it feels more personalised, and I think that’s just because you’re talking one to one on the telephone… And in the (face-to-face) clinic, sometimes people are popping in. I don’t know, you feel like in the (face-to-face) clinic there’s a bit more clock-watching going on because people are stressed and they’re having to deal with other situations other than just you.”* *“At the moment I am feeling pretty well, so I think that the phone one is absolutely fine. But I suspect that if I was struggling a bit, if I had a lot more symptoms, a lot more complications, I might feel that a face-to-face appointment would be better.”* *“The PrISMS clinic is a really good thing, because you can be interviewed in the comfort of your own home, and it takes away a lot of the tension of waiting around.”* *“I think it (PrISMS) was more inclusive which sounds a bit bizarre given it's remote, but I think sometimes it's easier to say things when you're just on your own to somebody on the telephone.”*
Holistic care	*“(The physiotherapist) took off and put me on the programme with the Tottenham Hotspurs physios, which was extremely useful getting a bit more active, a bit more movement. And building up some muscle power. That was absolutely invaluable.”* *“I don't even have to leave my home to get a more holistic service…and it was quite interactive with people chipping in and coming in and out of the conversation.”* *“I think the holistic nature of the consultations is much more beneficial. When you're dealing with just a consultant on a one-to-one basis it's, for me, a pretty narrow discussion.”*

Several patients specifically mentioned the benefits of having a PT in the clinic, who had motivated them to start exercising regularly either by themselves or through a local exercise scheme. Some also reported a positive impact on their mental health. However, some patients did not feel that they required PT input, either because they were already sufficiently active or because they were asymptomatic and did not have any specific physiotherapy needs. Similarly, some patients appreciated the additional input from CNS, whilst others felt that CNS was more needed when they were receiving active myeloma treatment or during an inpatient stay.

Opinions regarding remote monitoring varied: some preferred F2F clinic whilst others thought that remote clinic was more personal, had shorter waiting time and that they could wait to be reviewed in the comfort of their own homes. Furthermore, patients would like to have occasional F2F consultations in addition to remote monitoring. Overall, patients appreciated the MDT input and there was a sense of gratitude and desire for the pilot clinics to continue.

### Patient-reported outcome measures

The GLTEQ, MyPOS, EORTC QLQ-C30 and EORTC QLQ-MY20 scores of patients who had completed the questionnaires before their first and after their last PrISMS consultation are displayed in Table 3. There was a statistically significant improvement in GLTEQ scores post-PrISMS clinic, indicating an increase in PA (median score 20.0 versus 25.0, *p* = 0.02). There was no difference across all domains in the pre- and post-PrISMS clinic MyPOS and EORTC QLQ-C30 scores. There was a deterioration in the disease symptoms domain (median 17.0 versus 22.2, *p* = 0.02) and the body image domain (median 88.6 versus 100, *p* = 0.02) of the myeloma-specific EORTC QLQ-MY20 questionnaire post-PrISMS clinic. When relapsed patients were removed from the analysis, the difference in scores in the disease symptoms domain remained significant (*n* = 25, median 11.1 versus 22.2, *p* = 0.02) but not in the body image domain (*p* = 0.16).

**Table 3:** Pre- and post-PrISMS clinic PROM scores

	Pre-PrISMS clinic score			Post-PrISMS clinic score		Test	
Measure	No. of patients	Mean (SD)	Median (range)	Mean (SD)	Median (range)	Confidence Interval	*p*-value
*GLTEQ*	33	26.8 (30.2)	20.0 (0–160)	35.6 (29.6)	25.0 (0–101)	1.8–15.3	0.02*
*MyPOS*							
Total score	32	18.0 (17.2)	13.5 (0–83)	18.7 (14.0)	16.0 (0–57)	− 3.5–5.0	0.64
Symptoms	35	6.1(5.9)	5.0 (0–27)	5.5 (4.9)	4.0 (0–19)	− 2.0–1.0	0.42
Emotion response	33	11.0 (11.0)	7.0 (0–51)	12.0(9.2)	12.0 (0–35)	− 1.5–4.0	0.47
Healthcare support	33	1.0 (1.4)	0 (0–5)	1.0 (1.4)	0 (0–5)	− 0.5–0.5	0.93
*EORTC QLQ-C30*							
Global health status	35	75.0 (17.1)	83.3 (41.–100)	71.3 (19.6)	75.0 (33.3–100)	− 8.3–2.1	0.15
Physical function	37	80.4 (19.8)	86.7 (20–100)	79.0 (22.1)	86.7 (20–100)	− 3.3–3.3	0.65
Role function	37	80.2 (29.1)	100 (0–100)	81.1 (25.9)	100 (0–100)	0.0–8.3	0.78
Emotional function	35	77.4 (22.7)	83.3 (8.3–100)	80.6 (17.2)	83.3 (33.3–100)	− 4.2–8.3	0.36
Cognitive function	35	79.0 (27.2)	83.3 (0–100)	85.7(18.6)	100 (33.3–100)	0.0–8.3	0.09
Social function	35	80.5 (25.7)	100 (0–100)	76.2 (27.5)	83.3 (0–100)	− 8.3–0.0	0.21
*EORTC QLQ-MY20*							
Disease symptoms	31	17.0 (18.3)	11.1 (0–77.8)	22.1 (19.3)	22.2 (0–77.8)	0.0–8.3	0.02*
Side effects of treatment	31	15.1 (12.5)	10.0 (0–51.9)	12.4 (12.4)	11.1 (0–53.3)	− 5.6–0.2	0.14
Body image	35	88.6 (19.7)	100 (33.3–100)	79.0 (30.3)	100 (0–100)	− 16.7–0.0	0.02*
Future perspective	35	74.0 (22.9)	77.8 (22.2–100)	71.8 (23.5)	77.8 (0–100)	− 11.1–5.6	0.39

Values in bold indicate scores that are statistically different pre-PrISMS clinic and post-PrISMS clinic

## Discussion

Multidisciplinary outpatient clinics have been described in other specialities [19–21] with high levels of satisfaction. To our knowledge, this was the first MDT myeloma clinic that was implemented and evaluated prospectively. We demonstrated that PrISMS clinic was safe, with all relapsed patients referred promptly back to the F2F clinic. Patient compliance was high with excellent clinic attendance, and the majority completed their pre-clinic blood tests and questionnaires. Median call duration was acceptable at 13 min and included an MDT discussion with the patient covering clinical, psychosocial and PA topics. This was helped by the pre-clinic questionnaire, which clinicians reviewed in advance to ensure the effective use of clinic time. A notable outcome was the increase in physical activity post-PrISMS clinic, as evident in the significant increase in patients’ GLTEQ scores. We have previously shown that the majority of MM patients have low GLTEQ scores [11], which is demonstrated again here in the low pre-PrISMS GLTEQ scores. This underlines the low levels of exercise in our patients, likely due to existing myeloma bone complications and the fear of exacerbating these when exercising. However, the health benefits of PA are well described in cancer patients including those with MM [6, 7]. It has been shown to correlate significantly with social, functional, mental and sexual well-being, as well as lower pain and fatigue scores. We also demonstrated in our previous studies that higher exercise levels were associated with better QoL and that exercise intervention could improve muscle strength and cardiovascular fitness [9–11]. Moreover, the U.K. Independent Cancer Taskforce recommended in its strategy that all cancer patients should receive tailored PA advice. MM patients have expressed their desire to receive PA advice [11] and are more confident to exercise under the supervision of a physiotherapist experienced in working with myeloma [9, 10]. This is confirmed by the high percentage (90%) of PrISMS patients receiving PT advice during the study period. A small proportion of patients with more complex physiotherapy needs such as those with spinal involvement were offered additional remote or F2F PT sessions. Our experience in the PrISMS clinic indicates that PT input should be stratified based on clinical need, and advice can be delivered in a combination of ways including remote and F2F assessment, provision of individually tailored advice and signposting to existing resources or community schemes.

The unique MDT approach of PrISMS clinic is particularly relevant to myeloma, where patients often suffer from residual symptom burden despite having achieved a good response to therapy, persistent therapy-related side effects such as peripheral neuropathy, and age-related co-morbidities. Importantly, this clinic systematically covers aspects of management in the late or long-term consequences of myeloma and its treatment. A high proportion (80%) of participants received input from CNS, who promoted healthy lifestyle choices such as smoking cessation, a balanced diet and moderate alcohol consumption. Mental health issues have been shown to impact HRQOL directly and are a predictor of survival [5, 22–24]. Our CNS ensured that psychosocial issues were addressed, with referral to psychology or social services if appropriate. Through signposting to survivorship tools, patients were empowered to self-manage their condition, which is a key goal of the National Health Service’s UK Long Term Plan. Of note, two patients mentioned the need to include sexual activity in the pre-clinic questionnaire, highlighting an aspect of unmet survivorship need. Experience of sexuality-related issues is an important QoL concern among cancer survivors, but communication of such issues between patients and clinicians is often not routinely discussed in follow-up care [25].

It is worth mentioning that not all patients in the study required MDT input in every consultation and/or preferred remote monitoring. PrISMS clinic is well suitable for a selected patient cohort, including those with survivorship needs. We enrolled patients who were not on active myeloma treatment in the pilot clinics, as this would be a period when they could focus on returning to their pre-morbid functioning state. However, as the myeloma treatment paradigm evolves towards continuous and maintenance therapy, PrISMS clinic could be adapted to extend its benefit to clinically stable patients on maintenance treatment.

Our study did not demonstrate an improvement in HRQOL scores of the EORTC QLQ-C30, EORTC QLQ-MY20 and MyPOS questionnaires post-PrISMS clinics. This may be explained by the small sample size, and the fact that HRQOL and PA assessment were only performed at two time points. Importantly, the COVID-19 pandemic occurred during the study, which may have affected patients’ QoL and hence our PROM results. These HRQOL questionnaires were mostly validated against cancer or myeloma patients who were newly diagnosed or had relapsed disease and were on treatment [15, 26, 27]. They were not designed to assess the HRQOL of stable myeloma patients who are on observation with stable or few disease symptoms and hence may not be the best tools to assess the benefits of PrISMS clinic. On the contrary, qualitative data from patients’ testimonies suggest a clear positive impact on multiple aspects of patient care by the PrISMS clinic. There is, therefore, a need to focus beyond quantitative data and to develop better HRQOL assessment tools for myeloma survivorship.

PrISMS clinic was planned and launched before the COVID-19 pandemic. During the pandemic, it became apparent that its remote clinic design was ideal for reducing the risk of COVID-19 infection. Its holistic nature also provided patients with reassurance during the switch from F2F to remote appointments. Moreover, patients were able to benefit from other advantages of a remote clinic such as a reduction in travel time, cost and anxiety when waiting to see a doctor in the hospital. Overall, the clinic was well-received, with 95% of participants felt that their concerns were addressed and 83% rated good or excellent in their trust in the PrISMS team despite it being a remote clinic.

To conclude, PrISMS clinic offers a holistic MDT approach to myeloma outpatient care that is safe and feasible, featuring individualised management and patient empowerment. It provides bespoke guidance and promotes independent physical activity that patients find beneficial. It has high patient acceptability and can be used as a template for wider rollout nationally and internationally, as well as for other haematological subspecialties. In the aftermath of the COVID pandemic, where remote consultations are likely to remain a major feature of outpatient clinic models, the PrISMS clinic is an example of how we can deliver multidisciplinary consultations to provide more holistic care and support to cancer survivors.

## Supplementary Information

Below is the link to the electronic supplementary material.

Supplementary file1 (DOCX 135 KB)
