# Supplementary material for: High patient satisfaction and increased physical activity following a remote multidisciplinary team multiple myeloma clinic
Source: Support Care Cancer. 2023 Jan 21;31(2):127. doi: 10.1007/s00520-023-07587-9 (PMC9860216; doi:10.1007/s00520-023-07587-9)

**My Myeloma Review** – PrISMS clinic crib sheet

Name:……………………………………………………………………… Hospital number: ………………………………. Date: …………………………….

Please complete the questions which will be covered in your next telephone or video clinic appointment.

Prior to **every** appointment please update and email to: [uclh.prisms.myeloma@nhs.net](mailto:uclh.prisms.myeloma@nhs.net)

**Q1**. What are your main concerns at the moment relating to your Myeloma and general health?

**Q2**. Below is a list of **possible** symptoms which you **may** experience. For each symptom please consider how it has affected you since your last clinic appointment.

|  | **Not at all** | **Slightly** | **Moderately** | **Severely** | **Overwhelmingly** |
| --- | --- | --- | --- | --- | --- |
| Pain |  |  |  |  |  |
| Jaw pain or tooth ache |  |  |  |  |  |
| Difficulty breathing |  |  |  |  |  |
| Weakness or lack of energy |  |  |  |  |  |
| Nausea or feeling sick |  |  |  |  |  |
| Vomiting |  |  |  |  |  |
| Constipation |  |  |  |  |  |
| Diarrhoea |  |  |  |  |  |
| Poor appetite |  |  |  |  |  |
| Drowsiness |  |  |  |  |  |
| Reduced physical activity |  |  |  |  |  |
| Changes in mood |  |  |  |  |  |
| Tingling/numbness hands of feet, Jaw |  |  |  |  |  |

**Q3.** Is your myeloma or noted symptoms affecting your ability to return to your normal daily activities? If yes, which ones?

**Q4**. Have you had any infections since your **last** clinic appointment? Did you require any antibiotics?

**Q5**. Have you been admitted to hospital or had any new medical problems since your **last** appointment?

**Q6**. **Please list all medication you are talking at the moment ready for your clinic appointment, including occasional medication (e.g. pain killers, anti-sickness medication)**

**Q7.** How would you like to improve your health over the next 3 months?

**Q8.** During a typical 7-day period (a week), how many times on average do you do the following kinds of exercise:

| 1. STRENOUS EXERCISE (HEART BEATS RADIDLY – e.g. running, jogging, football, vigorous swimming, vigorous long distance bicycling) | Duration in minutes per episode: _____  Times per week: _____ |
| --- | --- |
| 1. MODERATE EXERCISE (NOT EXHAUSTING – e.g. fast walking, easy bicycling, easying swimming, dancing, volleyball, badminton) | Duration in minutes per episode: _____  Times per week: _____ |
| 1. MILD EXERCISE (MINIMAL EFFORT – e.g. yoga, fishing, bowling, golf, horse riding, easy walking) | Duration in minutes per episode: _____  Times per week: _____ |
| 1. During a typical 7-day period (a week), in your leisure time, how often do you engage in any regular activity **long enough to work up a sweat** (heart beats rapidly)? | Often  Sometimes  Never / Rarely |

**Q9.** Would you like to discuss about sexual activity? Yes  No

**Q10**. Do you have any other questions/advice you would like to discuss at your appointment?

**Evaluating Patient Reported Outcomes of Measures of the PrISMS clinic – Patient feedback questionnaire**

Participant ID number: _______________

Dear Patient,

Thank you for taking part in the Promoting Individualised Self-Management for Myeloma Survivorship (PrISMS) remote clinic.

We had a numbers of aims we wanted to achieve within the PrISMS clinic and we would be grateful for your answers on completing the patient experience survey.

Thank you,

Demographics:

**Gender: Months and years since diagnosis:**

| Male |  | Female |  |
| --- | --- | --- | --- |

|  | Years |  | Months |
| --- | --- | --- | --- |

**Marital status: Age:**

| 45yrs or under |  |
| --- | --- |
| 46 - 55 years |  |
| 56 - 65yrs |  |
| 66 - 75yrs |  |
| 76yrs or over |  |

| Single never married |  |
| --- | --- |
| Married/domestic partnership |  |
| Widowed |  |
| Divorced/Separated |  |

**Employment: Distance from UCLH:**

| Employed full-time |  |
| --- | --- |
| Employed part-time |  |
| Self-employed |  |
| Retired |  |
| Not employed |  |

| Under 10 Miles |  |
| --- | --- |
| 10- 20 miles |  |
| 21-30 miles |  |
| Over 30 miles |  |

| Yes | No |
| --- | --- |

**How do you travel to UCLH? Are you seen by a haematologist locally?**

| Public transport |  |
| --- | --- |
| Private car |  |
| Taxi car |  |
| Walk |  |
| Other |  |

| **Yes** | **No** |
| --- | --- |
|  |  |

Blood test:

**Were your blood results available for your consultation?**

**How useful did you find having the blood results available for the telephone consultation?**

| **1**  **Terrible** | **2**  **Poor** | **3**  **Average** | **4**  **Good** | **5**  **Excellent** |
| --- | --- | --- | --- | --- |
|  |  |  |  |  |

**How easy was it for you to have your bloods taken locally or at UCLH?**

| **1**  **Terrible** | **2**  **Poor** | **3**  **Average** | **4**  **Good** | **5**  **Excellent** |
| --- | --- | --- | --- | --- |
|  |  |  |  |  |

**Did you feel that you had enough time to get the blood tests done before the telephone clinic?**

| **1**  **Terrible** | **2**  **Poor** | **3**  **Average** | **4**  **Good** | **5**  **Excellent** |
| --- | --- | --- | --- | --- |
|  |  |  |  |  |

**Other comments:**

My Myeloma Review Questionnaire:

**How useful did you find the questions on the crib questionnaire for monitoring your own health?**

| **1**  **Terrible** | **2**  **Poor** | **3**  **Average** | **4**  **Good** | **5**  **Excellent** |
| --- | --- | --- | --- | --- |
|  |  |  |  |  |

**How easy was it to complete the crib questionnaire?**

| **1**  **Terrible** | **2**  **Poor** | **3**  **Average** | **4**  **Good** | **5**  **Excellent** |
| --- | --- | --- | --- | --- |
|  |  |  |  |  |

**How easy was it to receive crib questionnaire by email and return completed form back to the PrISMS email address?**

| **1**  **Terrible** | **2**  **Poor** | **3**  **Average** | **4**  **Good** | **5**  **Excellent** |
| --- | --- | --- | --- | --- |
|  |  |  |  |  |

| **Yes** | **No** |
| --- | --- |
|  |  |

**Do you feel the crib questionnaire improved the quality of your clinic appointment?**

**Other comments:**

Promoting Individualised Self-Management for Myeloma Survivorship:

**How well did the consultation discuss your clinical concerns?**

| **1**  **Terrible** | **2**  **Poor** | **3**  **Average** | **4**  **Good** | **5**  **Excellent** |
| --- | --- | --- | --- | --- |
|  |  |  |  |  |

| **Yes** | **No** |
| --- | --- |
|  |  |

**Was physical activity and fitness discussed in your telephone consultation?**

**Having a physiotherapist in the consultation was useful:**

| **1**  **Disagree strongly** | **2**  **Disagree** | **3**  **Neither agree nor disagree** | **4**  **Agree** | **5**  **Agree strongly** |
| --- | --- | --- | --- | --- |
|  |  |  |  |  |

**Having a nurse specialist in the consultation was useful:**

| **1**  **Disagree strongly** | **2**  **Disagree** | **3**  **Neither agree nor disagree** | **4**  **Agree** | **5**  **Agree strongly** |
| --- | --- | --- | --- | --- |
|  |  |  |  |  |

**How well did you feel your concerns and symptoms were addressed?**

| **1**  **Terrible** | **2**  **Poor** | **3**  **Average** | **4**  **Good** | **5**  **Excellent** |
| --- | --- | --- | --- | --- |
|  |  |  |  |  |

**Did you feel more confident in self managing your myeloma after the consultation?**

| **1**  **Terrible** | **2**  **Poor** | **3**  **Average** | **4**  **Good** | **5**  **Excellent** |
| --- | --- | --- | --- | --- |
|  |  |  |  |  |

**Is there any other information/advice you would like to be covered by the telephone clinic?**

**Other comments:**

The telephone clinic experience:

**How were you greeted in your telephone clinic?**

| **1**  **Terrible** | **2**  **Poor** | **3**  **Average** | **4**  **Good** | **5**  **Excellent** |
| --- | --- | --- | --- | --- |
|  |  |  |  |  |

**Time spent waiting for telephone consultation?**

| **1**  **Terrible** | **2**  **Poor** | **3**  **Average** | **4**  **Good** | **5**  **On time** |
| --- | --- | --- | --- | --- |
|  |  |  |  |  |

**How would you rate your feeling of confidence and trust in the PrISMS team (doctor, nurse and physiotherapist) via the telephone?**

| **1**  **No confidence** | **2**  **Poor** | **3**  **Average** | **4**  **Good** | **5**  **Excellent** |
| --- | --- | --- | --- | --- |
|  |  |  |  |  |

**Were you given time to ask questions?**

| **1**  **No time** | **2**  **Poor** | **3**  **Average** | **4**  **Good** | **5**  **As much time as needed** |
| --- | --- | --- | --- | --- |
|  |  |  |  |  |

**Did you feel involved in making decisions in your Myeloma care?**

| **1**  **Not involved** | **2**  **Poor** | **3**  **Average** | **4**  **Good** | **5**  **Excellent** |
| --- | --- | --- | --- | --- |
|  |  |  |  |  |

**Overall how do you rate your PRISMS telephone clinic experience compared to a face to face clinic?**

| **1**  **Terrible** | **2**  **Poor** | **3**  **Average** | **4**  **Good** | **5**  **Excellent** |
| --- | --- | --- | --- | --- |
|  |  |  |  |  |

**Other comments:**

**Evaluating PROMs of the PrISMS clinic – Telephone interview topic guide**

- **Greeting and introduction**

This interview should take up to 30 minutes. Interviewer should obtain verbal consent before proceeding (written consent for telephone interview already obtained when patients agreed to participate in this study).

- **Blood tests:**

1. How useful did you find having the blood results available for the telephone consultation?
   1. Please give details why you find it useful / not useful
2. How easy was it for you to have your bloods taken locally or at UCLH?
   1. If taken locally – was it easy to arrange with our blood request letter to GP / local doctor? What was the waiting time? Was it more convenient than coming to UCLH?
   2. If taken at UCLH – was it easy to come to UCLH for blood tests? What was the waiting time to for phlebotomy service? Was it more convenient than having them done locally (if not local to this area)? Was it more convenient than having bloods taken than wait for face-to-face clinic appointment?
3. Did you feel that you had enough time to get the blood tests done before the telephone clinic? (We recommended two to three weeks before appointment)
   1. If no – how much time should we allow to have blood tests done in your case?
4. Other comments regarding blood tests before clinic.

- **My Myeloma Review Questionnaire:**

1. How useful did you find the questions on the crib questionnaire for monitoring you own health?
   1. Which questions were particularly useful / not useful?
   2. Did it improve the quality of your clinic appointment? Please elaborate why it did or didn’t help.
2. How easy was it to complete the crib questionnaire?
   1. Were the questions clear and easy to answer?
   2. How long did it take for you to complete it? Was it too short / about right / too long?
3. How easy was it to receive crib questionnaire by email and return complete form back to the PrISMS email address?
   1. Was it better than sending by post?
   2. Did you use this email address for any other reasons? Were you aware that this email was not for clinical queries?
   3. Would you prefer more communication regarding your appointment via email in the future rather than phone calls or post?
4. Other comments regarding the My Myeloma Review crib sheet.

- **PrISMS telephone consultation:**

1. How well did the consultation discuss your clinical concerns?
   1. What was done well or not well?
   2. Did you feel that all your concerns and symptoms were discussed? Were they all addressed?
   3. Was there enough time to discuss everything?
2. Was having a physiotherapist in the consultation useful?
   1. Why was it useful or not useful?
   2. Did you follow any advice from the physiotherapist? Did it help with your symptoms? Did it impact on your quality of life?
3. Was having a nurse specialist in the consultation useful?
   1. Why was it useful or not useful?
   2. Did follow any advice from the nurse specialist? How did it impact you or your well-being?
4. Did you feel more confident in self managing your myeloma after the consultation?
   1. How did this compared with the usual face-to-face clinic?
   2. Did you feel that more or less was discussed in the telephone consultant regarding your health and well-being compared with face-to-face clinic?
5. Are there any other information / advice you would like to be covered by the telephone clinic? Please give details.
6. Other comments regarding the consultation itself.

- **The telephone clinic experience:**

1. How were you greeted in your telephone consultation?
   1. Were you able to hear the team clearly on the phone?
2. Did you spend a long time waiting for the telephone consultation?
   1. How did the waiting time compare with that of the face-to-face clinic?
3. How would you rate your feeling of confidence and trust in the PrISMS team via telephone?
   1. How does this differ with a face-to-face clinic?
4. Did you feel involved in making decisions in your myeloma care? Please give details.
5. Overall how do you rate your PrISMS telephone clinic experience compared to face-to-face clinic? Any other comments regarding your experience?

PrISMS clinic blood tests

- Full blood count
- Urea and electrolytes
- Liver function tests
- Bone profile
- Immunoglobulins
- Protein electrophoresis
- Serum free light chains

SUPPLEMENTARY TABLE 1 – Examples of survivorship resources

| ***Physical activity and exercise:***   - Myeloma UK Exercise Infosheet - https://www.myeloma.org.uk/documents/exercises-for-myeloma-patients-infosheet/ - NHS Choices exercise plans - <https://www.nhs.uk/live-well/exercise/strength-and-flex-exercise-plan/> - Blood Cancer UK: Blood Cancer and Keeping Active Page -<https://bloodcancer.org.uk/support-for-you/living-well/keeping-active/>   ***Health and wellbeing:***   - Untire Fatigue self-management mobile app - <https://www.nhs.uk/apps-library/untire-beating-cancer-fatigue/> - Mental health mobile apps - <https://www.nhs.uk/apps-library/category/mental-health/> - Macmillan Toilet card - <https://be.macmillan.org.uk/be/p-24952-macmillan-toilet-card.aspx>   ***Myeloma information:***   - Myeloma UK: Understanding myeloma - <https://www.myeloma.org.uk/understanding-myeloma/> - Macmillan Cancer Support: myeloma - https://www.macmillan.org.uk/cancer-information-and-support/myeloma |
| --- |

SUPPLEMENTARY FIGURE 1 - Feedback from patient surveys regarding pre-clinic blood tests and ‘My Myeloma Review’ questionnaire


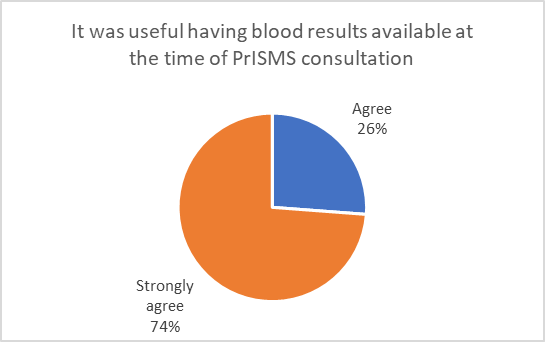

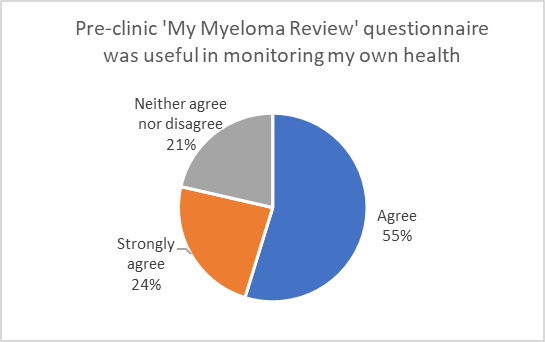

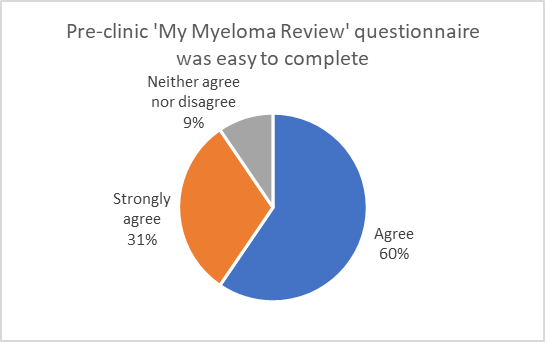


SUPPLEMENTARY FIGURE 2 - Feedback from patient surveys regarding PT and CNS input


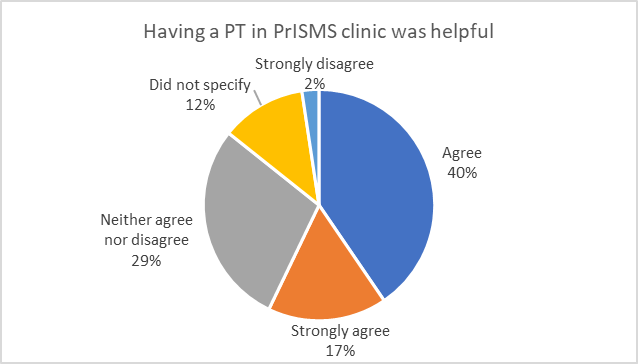

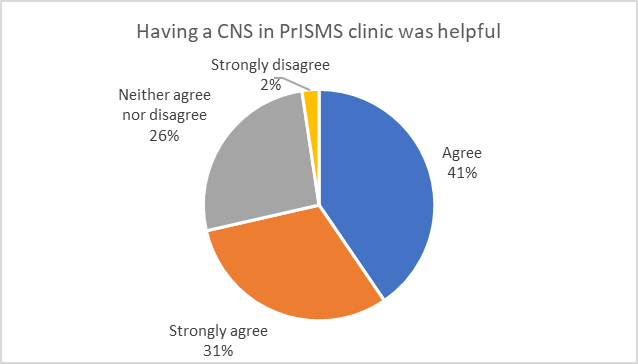

Supplement: Supplementary file 1 — Supplementary file1 (DOCX 135 KB) [file 520_2023_7587_MOESM1_ESM.docx]
